# Style-Semantic Disentangled Optical-to-Infrared Translation for Infrared Target Recognition

**DOI:** 10.3390/jimaging12080353

**Published:** 2026-08-03

**Authors:** Lizhuo Liu, Jiawei Niu, Lingxia Mu

**Affiliations:** 1 School of Electrical and Control Engineering, Xi’an University of Science and Technology, No. 58 Yanta Middle Road, Beilin District, Xi’an 710054, China; lizhuoliu@xust.edu.cn; 2 School of Electronic Engineering, Xidian University, No. 2 South Taibai Road, Yanta District, Xi’an 710071, China; 3 School of Automation and Information Engineering, Xi’an University of Technology, No. 5 Jinhua South Road, Xi’an 710048, China; mulingxia@xaut.edu.cn

**Keywords:** infrared target recognition, optical-to-infrared image translation, generative adversarial networks, style-semantic disentanglement

## Abstract

Infrared target recognition plays an important role in many real-world applications, but its performance is often constrained by the scarcity of annotated infrared data. To alleviate this issue, optical-to-infrared image translation has been widely explored as a data augmentation strategy by leveraging the abundance of optical images. However, existing approaches typically overlook the intrinsically multimodal nature of optical-to-infrared mapping, leading to insufficient diversity in the synthesized infrared images. Moreover, the lack of effective constraints to preserve semantic fidelity further hampers the practical utility of generated samples for recognition tasks. In this paper, we propose a multimodal style translation framework for infrared target recognition. The proposed framework is built upon a style-semantic disentanglement architecture, which decouples domain-general semantic structures from domain-specific style statistics, thereby enabling flexible recombination of optical content with diverse infrared characteristics. Furthermore, we design a multi-level adaptive loss function that explicitly enforces complementary constraints on structural fidelity and semantic consistency during the translation process. Extensive experiments on two public datasets demonstrate the effectiveness of SSD-VI. On RGB-NIR, it achieves an FID of 46.53 and a KID of 0.0331, while increasing classification accuracy by 6.68 percentage points, from 83.37% to 90.05%. On VEDAI, SSD-VI improves mAP@50 by 0.13 for YOLOv8m and 0.14 for RT-DETR, confirming the value of the generated samples for infrared target recognition.

## 1. Introduction

Infrared target recognition is a key capability in remote sensing and surveillance, as thermal imaging remains effective even in low illumination and adverse weather conditions [1,2,3]. Early studies were largely model-driven, relying on handcrafted features and statistical priors to separate targets from cluttered backgrounds [4]. With the advancement of deep learning, data-driven approaches have become dominant and have delivered substantial improvements [5,6,7], yet their performance is tightly coupled with the availability of large-scale annotated infrared data. In practice, acquiring and labeling infrared imagery is expensive and time-consuming, often resulting in insufficient supervision and, consequently, limited recognition performance.

Leveraging the complementarity between optical and infrared sensing, optical-to-infrared translation has been explored as a cost-effective means of augmenting infrared training data [8,9,10]. Most existing solutions are based on image-to-image (I2I) translation [11], commonly implemented with generative adversarial networks (GANs) [12], aiming to map images across domains while preserving semantic content. Depending on whether aligned training pairs are available, I2I translation can be categorized as paired or unpaired. Paired methods require strictly registered optical–infrared pairs, which are difficult to obtain in many practical scenarios [13]. Unpaired methods relax this requirement by introducing additional regularization to stabilize learning and are therefore more applicable in real-world settings [14]. In this work, we focus on unpaired optical-to-infrared translation for infrared data augmentation.

**Figure 1:**
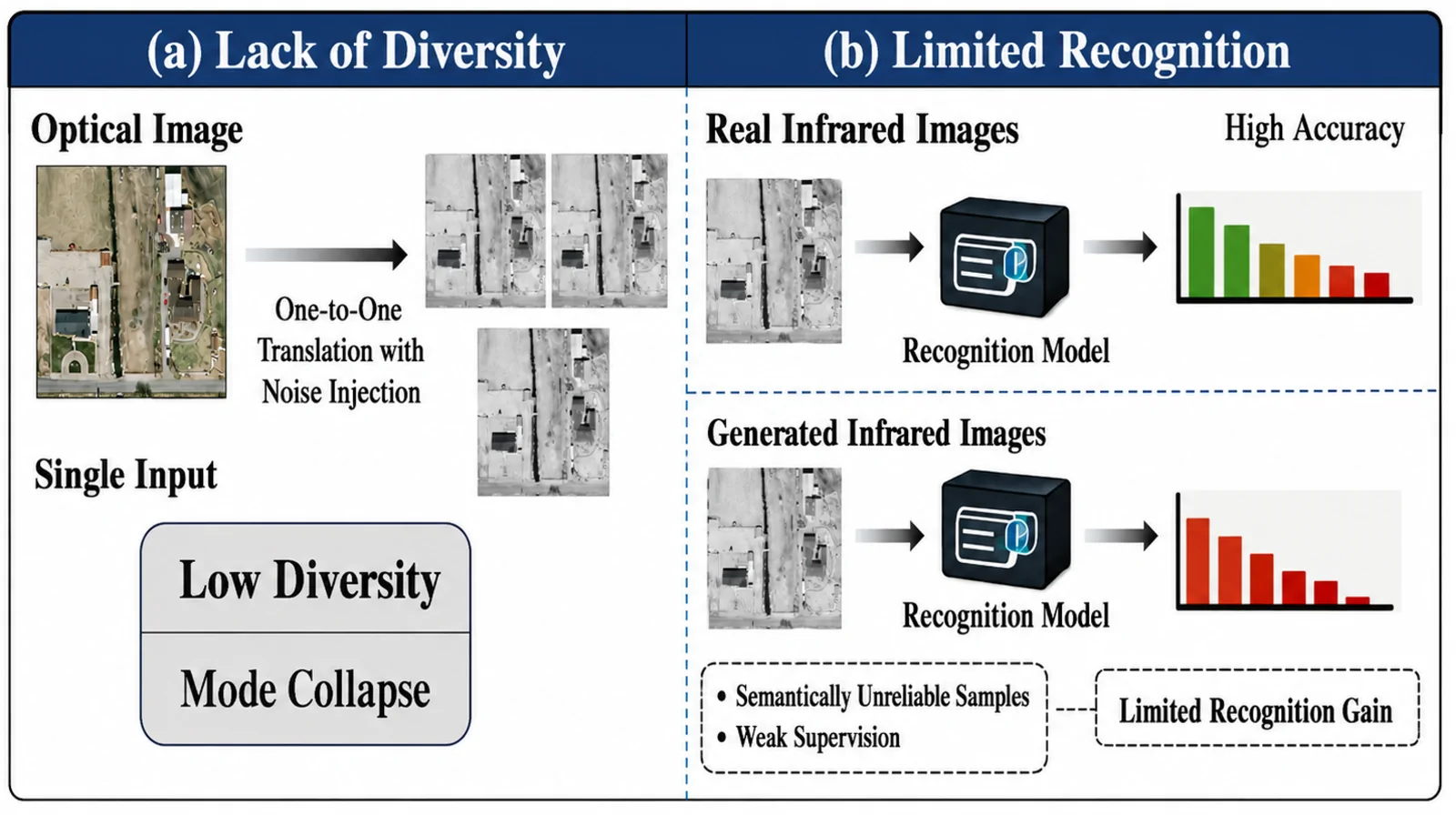
Key challenges in optical-to-infrared translation for infrared target recognition: (**a**) lack of appearance diversity due to the inherently multimodal cross-domain mapping; (**b**) limited recognition gains caused by semantically unreliable or weakly constrained synthetic samples.

Despite the progress in natural image translation, optical-to-infrared translation remains challenging when the generated images are intended for infrared target recognition. As summarized in Figure 1, two limitations are particularly critical. First, the correspondence between optical and infrared appearances is not strictly one-to-one. The infrared appearance of an object may vary with its thermal state, material properties, imaging conditions, and sensor characteristics, even when its optical structure remains similar. However, many existing translation methods learn a largely deterministic mapping or introduce diversity only through undirected noise injection. Such formulations provide limited control over infrared appearance variation and may generate highly similar outputs or suffer from mode collapse. This motivates the explicit separation of semantic structure from infrared-specific style, enabling multiple infrared appearances to be generated for the same optical content without altering the target identity or spatial layout. Second, visually plausible translation alone does not ensure that the generated images are useful for recognition. Conventional adversarial and cycle-consistency objectives mainly encourage domain realism and reconstruction consistency, but they do not explicitly prevent structural deformation or the loss of discriminative target information. Synthetic infrared images with a plausible global appearance may still contain distorted contours or weakened class-specific features, thereby limiting their value as training samples.

To address these issues, we propose SSD-VI, a style-semantic disentangled framework designed specifically for recognition-oriented optical-to-infrared data augmentation. The model retains modality-specific representations for low-level appearance modeling while aligning high-level semantic information shared across the optical and infrared domains. Infrared appearance variation is represented in a stochastic style space, enabling diverse outputs through style sampling and recombination rather than relying solely on unconstrained noise injection. Moreover, a multi-level adaptive objective constrains the generated images from complementary appearance and semantic perspectives. These design choices allow SSD-VI to generate infrared images that are not only diverse and visually plausible but also structurally consistent and semantically reliable for downstream target recognition. Comprehensive experiments demonstrate that our method generates diverse and semantically reliable infrared images, which effectively enhance data augmentation and improve infrared target recognition performance.

The contributions of this work are threefold. First, we propose SSD-VI, a multimodal translation framework specifically designed to bridge the semantic gap between optical and infrared domains. By generating diverse and high-fidelity infrared images, it effectively serves as a data augmentation strategy to improve recognition performance in data-scarce scenarios. Second, we construct a style-semantic disentangled network that explicitly addresses the ambiguity of optical-to-infrared mapping. By factorizing visual representations into domain-general semantics and domain-specific styles, our model enables style-conditioned generation of multiple infrared-domain appearances from a single optical input. Finally, we design a multi-level adaptive loss function that imposes complementary constraints across pixel, feature, and semantic levels. This ensures the synthesized images possess not only target-domain appearance statistics but also the discriminative features essential for accurate recognition.

The remainder of this paper is organized as follows. Section 2 introduces the related works briefly. Section 3 presents the proposed SSD-VI in detail. Section 4 describes the experimental settings and reports the experimental results. Section 5 presents the conclusions.

## 2. Related Work

In this section, we briefly review related work to our proposed method, including infrared target detection, optical-to-infrared image translation, and disentangled representation learning.

### 2.1. Infrared Target Recognition

Infrared target recognition methods can be broadly categorized into traditional model-driven approaches and data-driven deep learning-based approaches. Traditional model-driven methods typically rely on handcrafted features and manually tuned parameters derived from prior knowledge. Although such methods are effective in specific scenarios, their performance is often sensitive to parameter settings and environmental variations, resulting in limited robustness to complex and diverse infrared scenes. With advances in deep learning, data-driven methods have become the dominant paradigm for infrared target recognition. By leveraging large-scale training data, deep learning-based approaches can automatically learn discriminative representations through end-to-end optimization, resulting in improved adaptability and robustness. Among recognition tasks, infrared target detection has received substantial attention. To address the scarcity of infrared training data, Kim et al. [15] proposed a GAN-based framework that synthesizes infrared backgrounds from visible images and inserts realistic targets via intensity modulation for infrared small target detection. Li et al. [5] introduced a super-resolution YOLO-based framework that enhances infrared dim small target detection by incorporating coordinate attention, high-resolution feature fusion, and Swin Transformer blocks to improve robustness against noise, scale variation, and low contrast. Li et al. [16] further proposed a multibranch mutual-guiding learning network that jointly models target localization, edge information, and detection cues, thereby improving detection performance in cluttered infrared scenes. Yang et al. [17] designed a pinwheel-shaped convolution structure and scale-adaptive loss functions to address target size variations, enabling target-aligned receptive fields and dynamic weighting between scale and localization objectives. Zhang et al. [1] developed an interpretation-weighted sparse method that exploits target sparsity and local contrast cues within a multi-subspace modeling framework to suppress background clutter and enhance dim target responses.

Despite notable progress in deep learning-based infrared recognition methods, their performance still depends heavily on the availability of sufficient and diverse annotated infrared training data. In practical scenarios, infrared datasets often contain a limited number of targets and exhibit strong appearance variations due to changes in thermal conditions, materials, and imaging environments. As a result, models trained on scarce infrared data are prone to overfitting and may generalize poorly to unseen scenes. To address this problem, cross-modal data augmentation has emerged as a promising alternative. Given the abundance of optical images, translating optical data into infrared images provides an effective means of expanding infrared training sets. In particular, generating infrared images with diverse appearance styles while preserving target semantics can expose recognition models to a wider range of thermal variations.

### 2.2. Optical-to-Infrared Image Translation

Optical-to-infrared image translation aims to synthesize infrared images from optical inputs while preserving the underlying semantic structures. This task has attracted increasing attention due to its potential for augmenting infrared data, particularly in scenarios where annotated infrared data are scarce. Most existing approaches adopt generative adversarial networks (GANs) as the core framework, leveraging adversarial learning to align the distribution of generated images with that of real infrared data. Early studies primarily focused on general cross-domain generation. Larsen et al. [18] combined GANs with variational autoencoders to improve image reconstruction by learning more structured latent representations. Taigman et al. [19] proposed a domain transfer network with compound loss functions to enable cross-domain mapping. To relax the requirement of paired training data, Yi et al. [13] introduced DualGAN, which performs bidirectional translation through adversarial learning. Zhu et al. [14] further proposed CycleGAN by enforcing cycle-consistency constraints, making unpaired image-to-image translation practical and widely applicable.

Building upon these advances, image-to-image translation has been extensively studied across various application domains, including medical imaging, 3D content generation, and document understanding [20,21,22]. For instance, Kim et al. [23] developed a latent diffusion framework for multimodal medical image translation, while Zhang et al. [24] proposed a NeRF-to-NeRF translation model for flexible 3D scene editing. Wang et al. [25] introduced UniDIT to enhance document image translation by incorporating layout-aware representations. Nuanmeesri et al. [26] proposed Dendro-AutoCount to improve tree-ring measurement under local structural irregularities through pith localization and directional peak analysis, while MHA-PCNN [27] was developed to enhance visible–near-infrared feature interactions through parallel feature extraction and hybrid attention. Zhou et al. [28] proposed the SHIP to capture complementary cross-modal information through high-order interactions in both spatial and channel dimensions. Xiang et al. [29] proposed SimSID to detect and localize anomalies in radiographic images by exploiting recurrent anatomical structures through a space-aware memory matrix and feature-space inpainting.

More recently, diffusion models have demonstrated strong generative capability and stable distribution modeling in image-to-image translation. However, many diffusion-based translation methods rely on large-scale pretrained models or considerably higher training and sampling costs. These requirements differ from the limited-data, unpaired optical-to-infrared translation setting considered in this work. In addition, existing diffusion-based methods have rarely been developed specifically for recognition-oriented infrared data augmentation. A systematic comparison with diffusion-based methods under unified supervision and computational budgets, therefore, remains an important direction for future work.

Despite the progress of existing image translation methods, their direct application to optical-to-infrared data augmentation remains challenging. Most existing approaches formulate the translation as a deterministic one-to-one mapping, which fails to capture the inherently multimodal correspondence between optical and infrared domains. Moreover, commonly used adversarial and cycle-consistency constraints do not explicitly enforce semantic reliability, which limits the effectiveness of generated infrared images for target recognition tasks. These limitations motivate a translation framework that can model infrared appearance diversity while maintaining the semantic reliability required for downstream target recognition.

### 2.3. Disentangled Representation Learning

Disentangled representation learning aims to separate the underlying factors of variation in data into independent latent components, thereby enabling more interpretable, controllable, and diverse generation [30,31]. This approach has been widely explored across various vision tasks, including image generation and enhancement [32,33]. By decomposing image representations into content-related and attribute-related factors, disentangled models allow multiple plausible outputs to be generated from a single input. Several studies have demonstrated the effectiveness of disentangled representations in diverse application scenarios. Cheng et al. [34] proposed a VAE-based framework to learn disentangled instrumental variables for causal inference. Yin et al. [35] developed an unsupervised underwater image enhancement method that separates scene content from water-related degradations, improving generalization across environments. Xu et al. [36] introduced a controllable person image generation framework based on disentangled latent representations with transformer-based encoders.

Inspired by these advances, disentangled representation learning has also been introduced into image-to-image translation to mitigate mode collapse and improve generation diversity. Disentanglement itself is not a new concept, and many existing frameworks separate shared content from domain-specific appearance. However, most of these methods are developed for natural image translation and primarily emphasize control of visual appearance, without explicitly accounting for the preservation of recognition-relevant information. The distinction of SSD-VI lies in its recognition-oriented formulation for optical-to-infrared data augmentation. Specifically, it retains modality-specific low-level encoders to accommodate the substantial appearance differences between optical and infrared images, while aligning high-level semantic representations shared across the two modalities. Infrared-domain variations are modeled through stochastic style sampling, and the generated images are further constrained at the appearance, structural, and semantic levels. Therefore, the objective of SSD-VI is not merely to produce visually diverse infrared-like images, but to generate samples that preserve the semantic information required for downstream infrared target recognition.

## 3. Style-Semantic Disentanglement Representation Learning for Optical-to-Infrared Translation

**Figure 2:**
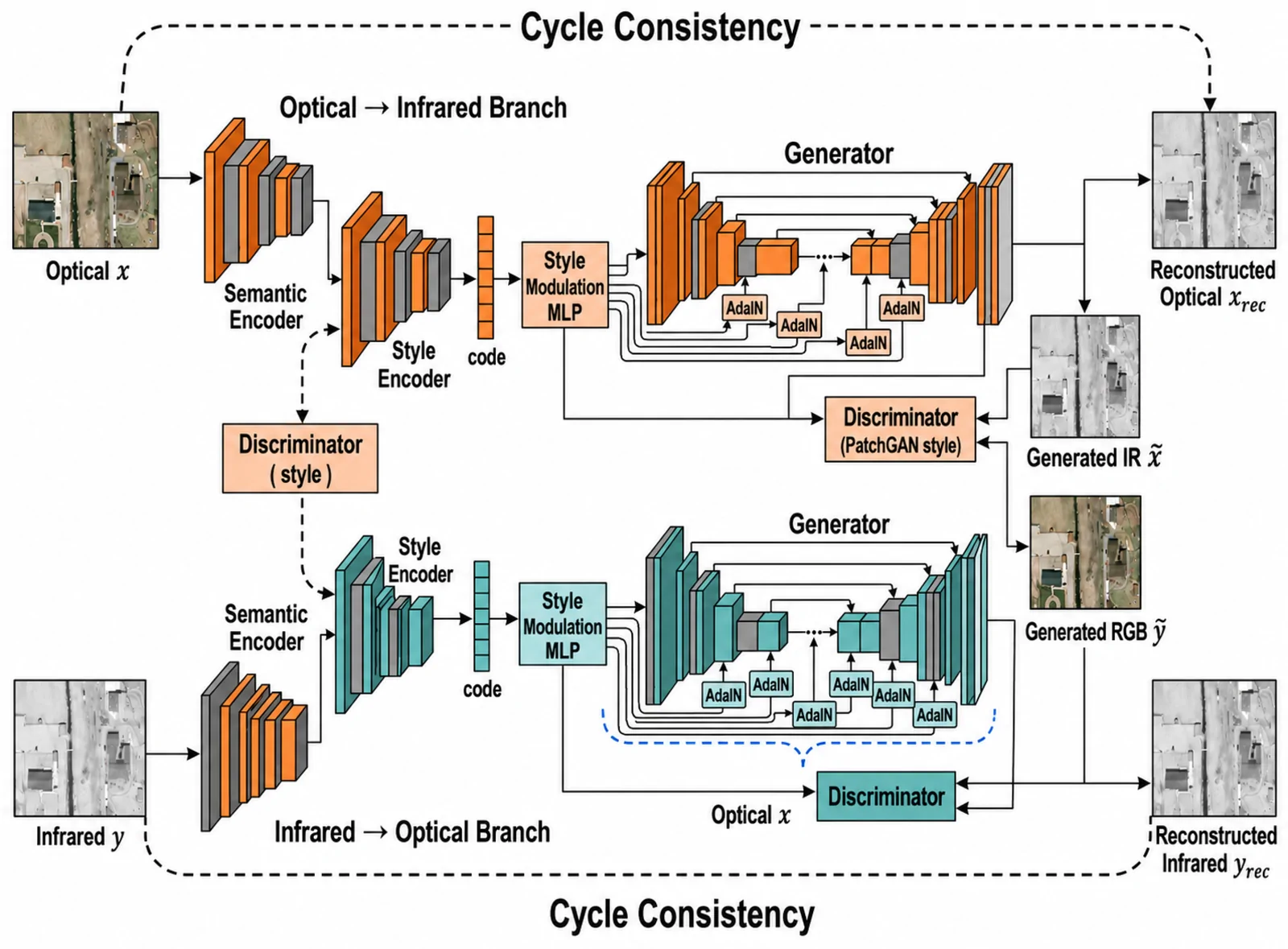
The overview of the structure of SSD-VI.

SSD-VI is a recognition-oriented optical-to-infrared translation framework designed to augment infrared training data and thereby improve infrared target recognition under limited data conditions. The framework aims to generate diverse and semantically reliable infrared images from optical inputs, ensuring that the synthesized samples are effective for recognition tasks. As illustrated in Figure 2, SSD-VI is built upon the CycleGAN architecture and is extended with a style-semantic disentanglement mechanism and multimodal supervision. Specifically, SSD-VI comprises semantic encoders, style encoders, cross-domain generators, and multiple discriminators. The semantic encoders project optical and infrared images into a shared domain-general semantic space, while the style encoders extract domain-specific style representations. Based on the disentangled representations, the cross-domain generators synthesize translated images by combining semantic features with randomly sampled style features during forward translation and reconstruct the original images during the cycle-consistency process. To facilitate effective domain alignment, a domain discriminator is employed to distinguish between optical and infrared domains, while adversarial discriminators are used to differentiate real images from generated ones.

### 3.1. Style-Semantic Disentanglement Representation

Our framework adopts a style-semantic disentangled representation to explicitly separate domain-general semantic content from domain-specific appearance variations. To this end, optical and infrared images are projected into a shared semantic space and two independent style spaces, enabling flexible recombination for multimodal optical-to-infrared translation.

Given an optical image x∈X and an infrared image y∈Y, semantic features are first extracted by the corresponding semantic encoders:

(1) $$c_{x}=E_{x}^{sem}\left(x\right),\;c_{y}=E_{y}^{sem}\left(y\right),$$

where $c_{x}$ and $c_{y}$ represent domain-general semantic representations that preserve structural and content information shared across modalities.

To encourage semantic consistency across modalities, the optical and infrared semantic encoders are designed to partially share their parameters. Optical and infrared images exhibit substantial differences in their low-level appearance statistics. Therefore, the early layers of the two encoders are kept modality-specific to capture domain-dependent cues, such as visible-spectrum texture and reflectance in optical images and intensity-contrast patterns in infrared images. Fully sharing the encoders may impose overly restrictive constraints on low-level feature learning, potentially leading to negative transfer between the two modalities. In contrast, higher-level features generally encode more modality-invariant information related to object structure, spatial layout, and semantic identity. We therefore share only the final layer of the semantic encoders, which promotes the alignment of high-level representations in a common latent semantic space while preserving modality-specific feature extraction in the preceding layers.

In addition to partial parameter sharing, a semantic-domain discriminator $D_{\mathrm{sem}}$ is introduced to further reduce the modality discrepancy between the high-level semantic representations. It takes $c_{x}$ and $c_{y}$ as inputs and predicts whether each representation originates from the optical or infrared domain. Through adversarial optimization, the semantic encoders are encouraged to produce domain-aligned representations that retain structural and semantic information while suppressing modality-specific appearance cues.

In parallel, domain-specific appearance information is encoded by the style encoders $E_{x}^{sty}$ and $E_{y}^{sty}$. Each style encoder predicts the mean and standard deviation of a $d_{s}$-dimensional Gaussian distribution, from which a latent style vector is sampled using the reparameterization trick:

(2) $$\left\{\begin{array}{cc} \left(\mu_{x},\sigma_{x}\right) & =E_{x}^{sty}\left(x\right),\;z_{x}=\mu_{x}+\sigma_{x}⊙\epsilon_{x},\;\epsilon_{x}∼N\left(0,I_{d_{s}}\right),\\ \left(\mu_{y},\sigma_{y}\right) & =E_{y}^{sty}\left(y\right),\;z_{y}=\mu_{y}+\sigma_{y}⊙\epsilon_{y},\;\epsilon_{y}∼N\left(0,I_{d_{s}}\right), \end{array}\right.$$

where $\mu_{x},\sigma_{x},z_{x},\mu_{y},\sigma_{y},z_{y}\in R^{d_{s}}$, and $d_{s}$ denotes the dimensionality of the latent style space. In all experiments, $d_{s}$ is set to 8. The noise vectors $\epsilon_{x}$ and $\epsilon_{y}$ are independently sampled from the standard normal distribution. To regularize the style space and facilitate continuous sampling, a KL-divergence loss is applied to encourage the learned latent distributions to approach a standard normal prior. Specifically, the KL-divergence regularization is defined as

(3) $$\begin{array}{cc} L_{\mathrm{KL}}= & \;E_{x∼X}\left[D_{\mathrm{KL}}\left(q_{x}\left(z_{x}|x\right)\;∥\;N\left(0,I\right)\right)\right]\\ \; & +E_{y∼Y}\left[D_{\mathrm{KL}}\left(q_{y}\left(z_{y}|y\right)\;∥\;N\left(0,I\right)\right)\right]. \end{array}$$

The sampled style vectors are subsequently transformed by a single shared multilayer perceptron (MLP) into the affine parameters required by adaptive instance normalization (AdaIN):

(4) $$\left(\gamma_{x},\beta_{x}\right)=\mathrm{MLP}\left(z_{x}\right),\;\left(\gamma_{y},\beta_{y}\right)=\mathrm{MLP}\left(z_{y}\right),$$

where $\gamma_{x},\beta_{x},\gamma_{y},\beta_{y}\in R^{C}$, and *C* denotes the number of feature channels. The shared MLP maps each $d_{s}$-dimensional style vector to 2C channel-wise affine parameters.

During cross-domain translation, semantic content and style features from different domains are recombined through AdaIN. Specifically, infrared-style features are applied to optical semantic representations to synthesize pseudo-infrared features, while optical-style features are combined with infrared semantic representations for the reverse mapping:

(5) $$\left\{\begin{array}{cc} f_{y}^{\prime } & =\mathrm{AdaIN}\left(c_{x},\gamma_{y},\beta_{y}\right)=\gamma_{y}\times \frac{c_{x}-\mu \left(c_{x}\right)}{\sigma (c_{x})}+\beta_{y},\\ f_{x}^{\prime } & =\mathrm{AdaIN}\left(c_{y},\gamma_{x},\beta_{x}\right)=\gamma_{x}\times \frac{c_{y}-\mu \left(c_{y}\right)}{\sigma (c_{y})}+\beta_{x}. \end{array}\right.$$

Two cross-domain generators are employed to perform bidirectional translation:

(6) $$\tilde{x}=G_{X}\left(f_{x}^{\prime }\right),\;\tilde{y}=G_{Y}\left(f_{y}^{\prime }\right),$$

where $\tilde{y}$ denotes the generated infrared image synthesized from optical semantics and infrared styles, and $\tilde{x}$ represents the reconstructed optical image in the reverse direction. This design ensures that domain-general semantics are preserved while adapting to target-domain appearance characteristics.

Unlike the semantic-domain discriminator, which operates on latent representations, the image-domain discriminators $D_{X}$ and $D_{Y}$ operate directly on images. Specifically, $D_{X}$ distinguishes real optical images from images translated into the optical domain, whereas $D_{Y}$ distinguishes real infrared images from images translated into the infrared domain. These two discriminators encourage the generated images to follow the appearance distributions of their respective target domains. For optical-to-infrared translation, the adversarial objective is defined as:

(7) $$L_{adv}^{Y}=E_{y∼Y}\left[\log D_{Y}\left(y\right)\right]+E_{\tilde{y}∼Y}\left[\log\left(1-D_{Y}\left(\tilde{y}\right)\right)\right],$$

and similarly for the infrared-to-optical direction:

(8) $$L_{adv}^{X}=E_{x∼X}\left[\log D_{X}\left(x\right)\right]+E_{\tilde{x}∼X}\left[\log\left(1-D_{X}\left(\tilde{x}\right)\right)\right].$$

The semantic-domain classification loss is formulated as

(9) $$L_{\mathrm{dom}}=-E_{x∼X}\left[\log D_{\mathrm{sem}}\left(c_{x}\right)\right]-E_{y∼Y}\left[\log\left(1-D_{\mathrm{sem}}\left(c_{y}\right)\right)\right].$$

### 3.2. Cycle-Consistency Constraint

To preserve semantic coherence during cross-domain translation, SSD-VI incorporates a cycle-consistency constraint following the CycleGAN paradigm. Cycle consistency enforces that an image translated to the opposite domain and then mapped back should reconstruct the original input, thereby preventing arbitrary content distortion during translation.

Specifically, the infrared-to-optical generator $G_{X}$ maps infrared semantics with optical styles to the optical domain, while the optical-to-infrared generator $G_{Y}$ synthesizes infrared images from optical semantics and infrared styles. During training, the two generators are applied sequentially to form a closed translation cycle. The cycle-consistency loss is defined as:

(10) $$\begin{array}{cc} L_{\mathrm{cyc}}= & \;E_{x∼X}\left[{∥G_{X}\left(G_{Y}\left(x\right)\right)-x∥}_{1}\right]\\ \; & +E_{y∼Y}\left[{∥G_{Y}\left(G_{X}\left(y\right)\right)-y∥}_{1}\right], \end{array}$$

which encourages the translated images to remain semantically faithful to the original inputs.

### 3.3. Multi-Level Alignment Loss

While the adversarial objective encourages the translated images to follow the target-domain appearance distribution and the cycle-consistency constraint discourages substantial content changes, neither objective explicitly preserves recognition-relevant structural and semantic information. We therefore introduce a multi-level alignment loss (MAL) that comprises perceptual structure alignment and semantic consistency constraints. Together, these constraints improve the reliability of the generated infrared images for downstream recognition.

#### 3.3.1. Perceptual Structure Constraints

Optical images provide rich structural details such as edges, contours, and local textures, which are important for preserving object geometry during translation. To prevent structural degradation, we impose a perceptual constraint between real optical images *x* and their reconstructed counterparts $\tilde{x}$ in a mid-level feature space. The perceptual loss is defined as:

(11) $$L_{\mathrm{perc}}=E_{x,\tilde{x}∼X}\left[∥\phi_{l}\left(x\right)-\phi_{l}\left(\tilde{x}\right){∥}_{1}\right],$$

which encourages the preservation of fine-grained structural information during translation.

#### 3.3.2. Semantic Consistency Constraints

To ensure that translated images remain reliable for recognition, high-level semantic consistency across modalities is explicitly enforced. The shared semantic encoder $E^{\mathrm{sem}}$ extracts domain-general representations from both real and generated images, capturing object-level and structural semantics. The semantic consistency loss is defined as:

(12) $$\begin{array}{cc} L_{\mathrm{sem}}= & \;E_{x∼X,\;\tilde{y}∼Y}\left[\;∥E^{\mathrm{sem}}\left(x\right)-E^{\mathrm{sem}}\left(\tilde{y}\right){∥}_{1}\;\right]\\ \; & +E_{y∼Y,\;\tilde{x}∼X}\left[\;∥E^{\mathrm{sem}}\left(y\right)-E^{\mathrm{sem}}\left(\tilde{x}\right){∥}_{1}\;\right]. \end{array}$$

which enforces semantic alignment between original images and their translated counterparts.

The complete training objective of SSD-VI is formulated as

(13) $$L_{\mathrm{total}}=\lambda_{\mathrm{adv}}L_{\mathrm{adv}}+\lambda_{\mathrm{cyc}}L_{\mathrm{cyc}}+\lambda_{\mathrm{perc}}L_{\mathrm{perc}}+\lambda_{\mathrm{sem}}L_{\mathrm{sem}}+\lambda_{\mathrm{KL}}L_{\mathrm{KL}}+\lambda_{\mathrm{dom}}L_{\mathrm{dom}},$$

where $L_{\mathrm{adv}}=L_{\mathrm{adv}}^{X}+L_{\mathrm{adv}}^{Y}$.

## 4. Experiments

In this section, we conduct comprehensive experiments to evaluate the effectiveness of SSD-VI. First, we introduce the experimental setup, including datasets, evaluation metrics, compared methods, and implementation details. Then, we present experimental results and analyses from two perspectives: quantitative and qualitative comparisons of optical-to-infrared translation quality, and infrared target recognition performance using the generated images for data augmentation. Finally, we provide ablation and parameter studies to further investigate the contribution of each component and the robustness of SSD-VI.

### 4.1. Experimental Setup

#### 4.1.1. Dataset

We validate the proposed SSD-VI on two public datasets for optical-to-infrared image translation: RGB-NIR [37] and VEDAI [38]. The details of the datasets are described below.

**Figure 3:**
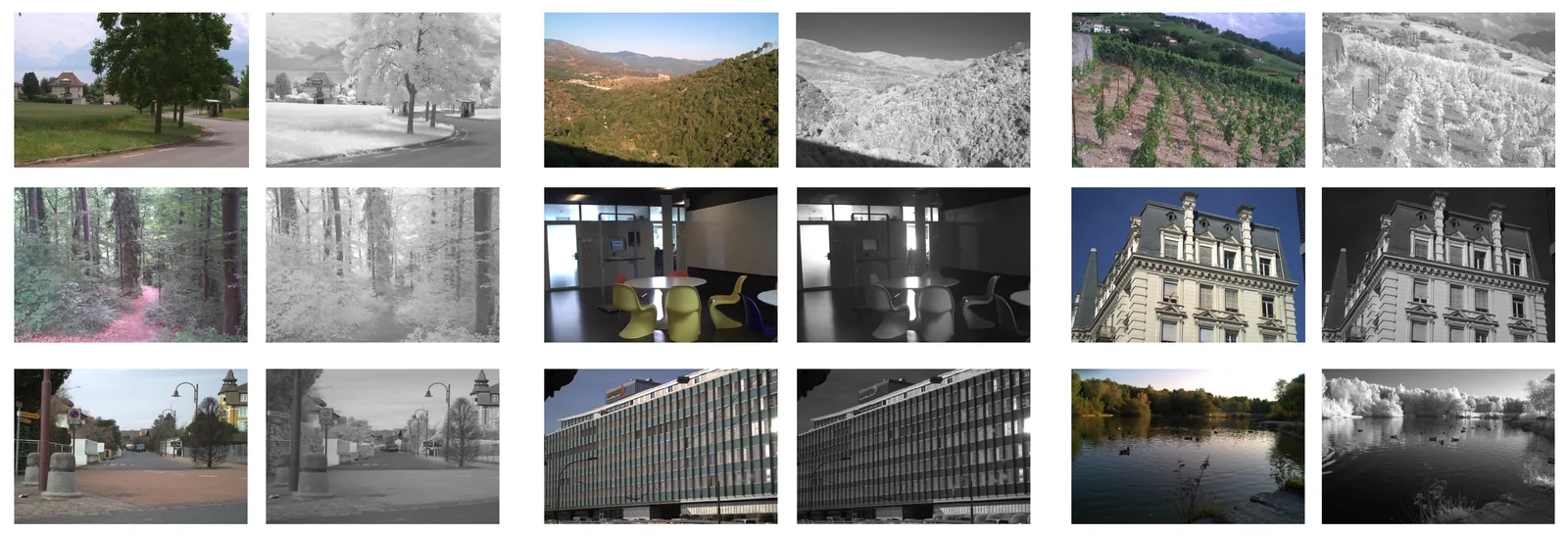
Instances of paired optical and infrared images of the RGB-NIR dataset.

The RGB-NIR dataset [37] contains 477 paired optical (RGB) and infrared (near-infrared) images collected from nine scene categories, including country, mountain, field, forest, indoor, old building, street, urban, and water. Representative samples from each category are illustrated in Figure 3. All images are resized to 224×224 pixels using bilinear interpolation. Since the infrared images are single-channel, they are replicated to three channels to match the input format of the network.

**Figure 4:**
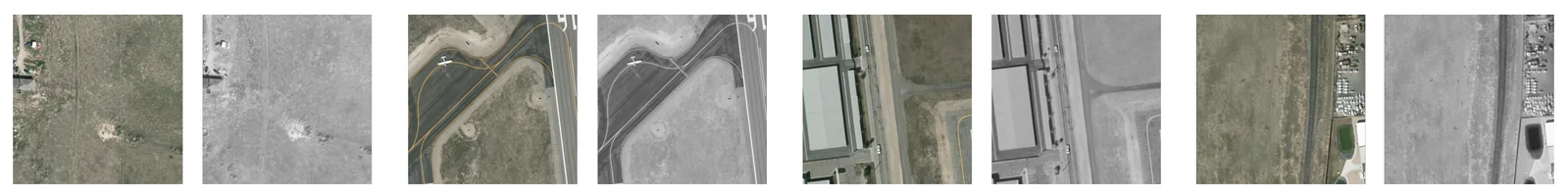
Instances of paired optical and infrared images of the VEDAI dataset.

The VEDAI dataset [38] is collected over Utah, USA, and covers a wide range of rural, agricultural, and urban environments, such as roads, residential areas, city blocks, barren lands, construction sites, and fields. It includes nine object categories—plane, camping car, tractor, car, truck, boat, van, pick-up, and an additional “other” class. Sample optical–infrared image pairs are shown in Figure 4. Two image resolutions are provided in VEDAI: 512×512 and 1024×1024. In our experiments, we use the VEDAI 1024 subset, in which the first half of the images is used for training and the remaining half for testing.

#### 4.1.2. Evaluation Metrics

We evaluate the proposed SSD-VI from two complementary perspectives: the quality of the generated infrared images and their effectiveness as augmented data for downstream infrared recognition tasks. For image translation quality, we employ FID, KID, LPIPS, and SSIM. FID and KID assess the distributional discrepancy between generated and real infrared images, whereas LPIPS and SSIM characterize perceptual diversity and structural consistency, respectively. These metrics provide complementary evidence regarding whether the synthesized images resemble the target infrared domain while preserving the content of the corresponding optical inputs. To evaluate the usefulness of the synthesized images for infrared target classification, we conduct experiments on the RGB-NIR dataset by training the classifier with both real and generated infrared images. Classification performance is evaluated using accuracy, recall, and F1-score. We further assess the practical value of the generated images for infrared target detection on the VEDAI dataset. The synthesized infrared images are incorporated into the detector training set, and the resulting performance is reported in terms of mAP@50, precision, and recall.

Image translation quality

FID and KID are jointly adopted to measure the discrepancy between the deep-feature distributions of generated and real infrared images. Both metrics are computed using features extracted by an Inception-V3 network, but they characterize distributional similarity through different formulations. FID approximates the two feature distributions as multivariate Gaussian distributions and measures their distance based on the corresponding means and covariance matrices. KID instead estimates the squared maximum mean discrepancy between the two feature sets using a polynomial kernel, without requiring an explicit Gaussian assumption. Lower values of both metrics indicate that the generated infrared images are closer to the real infrared distribution. Generation diversity is evaluated by computing LPIPS and SSIM among multiple infrared images generated from the same optical input under different style samplings. LPIPS measures perceptual differences using deep feature distances, with higher values indicating greater perceptual diversity. SSIM measures structural similarity at the pixel level, with lower values indicating larger structural variations across generated samples.

Target recognition performance

We use accuracy, recall, and F1-score to evaluate classification performance. Accuracy is the proportion of correctly classified test samples. Recall measures the proportion of ground-truth samples correctly recognized. The F1-score provides a balanced evaluation by jointly considering precision and recall. Higher values of these metrics indicate stronger discriminative capability introduced by the synthesized infrared data.

Detection performance is evaluated using mAP@50, precision, and recall. mAP@50 computes the mean average precision over all categories at an IoU threshold of 0.5, reflecting detection accuracy under moderate localization tolerance. Precision indicates the proportion of correctly localized targets among all predicted bounding boxes, while Recall measures the proportion of ground-truth targets that are successfully detected. Higher precision and recall imply improved robustness against false alarms and enhanced target-detection capability in complex infrared scenes.

#### 4.1.3. Compared Methods

We compare the proposed SSD-VI with several representative image-to-image translation methods, including general-purpose baseline and infrared-related approaches, to ensure a comprehensive and fair evaluation.

General-purpose image-to-image translation methods

Pix2Pix [39] is a representative paired image-to-image translation framework and serves as a supervised baseline for cross-domain mapping. CycleGAN [14] extends adversarial learning to unpaired settings by introducing cycle-consistency constraints. Following common practice, random noise is injected into the generators to enable stochastic generation.

Infrared-related and domain-aware methods

DSMAP [40] adopts domain-specific mappings from a shared content space to model cross-domain appearance variations. InfraGAN [41] explores infrared image synthesis by enhancing the capacity of the generator–discriminator framework. IR-GAN [42] further incorporates structure-aware constraints, such as SSIM-based loss and gradient regularization, to better preserve edges and structural details in translated infrared images.

Recognition models

YOLO-CIR [43] is an infrared-oriented object detection model that enhances YOLO with ConvNeXt-based feature extraction and attention mechanisms. DOCI-GAN [44] adopts a conditional inpainting framework to synthesize infrared multi-class object images for detection. TC-GAN [45] employs temperature-aware conditioning to generate diverse infrared images from visible inputs. IMT [46] uses a CycleGAN-based translation network to generate cross-modality images for visible–infrared recognition. ILoFGAN [47] introduces a local fusion GAN with attention-enhanced generators for data synthesis with an extremely limited number of samples. DTAGAN [48] adopts an attention-guided Wasserstein GAN with gradient penalty and dual-threshold training to generate infrared images. CWGAN-GP [49] with a residual network employs a conditional Wasserstein GAN with gradient penalty and residual blocks for infrared image synthesis.

#### 4.1.4. Experimental Settings

All experiments are conducted on a workstation equipped with an Intel Xeon Silver 4210R CPU and an NVIDIA GeForce RTX 3080 Ti GPU. The models are implemented usingPyTorch 1.13.1 with Python 3.9, and CUDA 11.2 is used for GPU acceleration.

We adopt the Adam optimizer for training, with a learning rate of $2\times 10^{-4}$, $\beta_{1}=0.5$, and $\beta_{2}=0.999$. The model parameters from the final training epoch are used for evaluation. The loss coefficients are selected using a held-out validation subset, without using the test data for hyperparameter selection. We fix $\lambda_{\mathrm{adv}}=1$ as the reference weight and initialize the remaining coefficients according to commonly used settings in image translation. Representative values within a local neighborhood of these initial settings are then examined, and the coefficients are adjusted in a staged manner rather than through an exhaustive grid search. The final configuration is set to $\lambda_{\mathrm{cyc}}=5$, $\lambda_{\mathrm{perc}}=0.5$, $\lambda_{\mathrm{sem}}=2$, $\lambda_{\mathrm{KL}}=0.01$, and $\lambda_{\mathrm{dom}}=0.1$, based on a joint consideration of FID and recognition performance on the validation subset. The same configuration is used for all datasets and experiments.

To ensure a fair comparison, all image translation methods are trained using the same training split and input resolution, and each method is used to generate the same number of synthetic infrared images. For downstream evaluation, the real infrared training subset, the ratio of synthetic to real samples, the classifier or detector architecture, the optimization schedule, and the test set are kept identical across all augmentation methods.

During inference, multiple infrared images are generated for each input optical image by randomly sampling style representations. FID and KID are computed by comparing all generated infrared images with the corresponding real infrared images. For diversity evaluation, LPIPS and SSIM are calculated over five randomly sampled pairs of generated infrared images for each input. For recognition tasks, the generated infrared images are combined with the available real infrared data to construct augmented training sets for classification and detection models, while evaluation is performed on real infrared test data. Each experiment is repeated five times under the same settings, and the reported results are averaged over the five runs.

### 4.2. Experimental Results and Discussion

#### 4.2.1. Quantitative Results

We quantitatively evaluate the proposed SSD-VI on two benchmark datasets using metrics that characterize distributional realism (FID and KID) and inter-sample variation (LPIPS and SSIM). The results are reported in Table 1 and Table 2. As shown in the tables, SSD-VI achieves favorable overall results on both datasets. In particular, the proposed method consistently obtains lower FID and KID scores, indicating that the generated infrared images are closer to the real infrared data distribution. At the same time, SSD-VI yields higher LPIPS and lower SSIM values among images generated from the same optical input, reflecting greater appearance variation under different style samplings. LPIPS and inter-sample SSIM should not, however, be interpreted independently as measures of training-data quality. These metrics quantify the degree of variation among generated samples, but do not determine whether the variations preserve target identity or remain useful for recognition. We therefore evaluate the practical utility of the synthetic data through downstream classification and detection on real infrared test images, as reported later in Table 3 and Table 4. The observed results can be attributed to two main design choices. First, the proposed style-semantic disentanglement separates domain-general semantic representations from domain-specific infrared styles, enabling the generation of different infrared appearances from the same optical input. Second, the multi-level alignment loss imposes complementary constraints at the style, structural, and semantic levels to reduce undesirable distortions in appearance and content during translation.

Table 3 reports the object detection results on the VEDAI dataset under a data-scarce setting, where only 60% of the real infrared images are used for training. The baseline method is trained without any data augmentation.

Across both YOLOv8m and RT-DETR detectors, all augmentation-based methods consistently outperform the baseline, confirming the effectiveness of synthetic infrared data for enhancing detection performance. For YOLOv8m, the proposed method achieves the best overall results, improving mAP@50 from 0.51 to 0.64, together with notable gains in both precision and recall. For RT-DETR, a similar performance trend is observed: although TC-GAN achieves the highest precision, it suffers from lower recall, resulting in inferior mAP. In contrast, the proposed method yields the highest mAP@50 and recall, indicating a more balanced trade-off between detection accuracy and target coverage. Overall, the consistent improvements across two detectors demonstrate that the proposed method can enhance detection performance.

To evaluate cross-dataset generalization, SSD-VI is trained exclusively on the VEDAI training set and directly evaluated on the RGB-NIR test set without fine-tuning. As reported in Table 5, SSD-VI obtains an FID of 60.0824 and a KID of 0.0612 in this setting. Compared with the in-dataset evaluation, the larger distribution discrepancies indicate that the model can generate infrared-domain outputs for previously unseen data, but its ability to match the target distribution is affected by differences in scene content, spectral characteristics, and sensor response. We further evaluate the robustness of SSD-VI to changes in input resolution. The model is trained using VEDAI images at a resolution of 512×512 and then evaluated on test images at resolutions of 512×512 and 1024×1024. When the test resolution increases to 1024×1024, the FID increases from 32.0845 to 39.7381, while the KID increases from 0.0071 to 0.0084. This moderate degradation suggests that SSD-VI can accommodate changes in spatial resolution to some extent, although its distribution-matching performance remains sensitive to resolution shifts.

**Figure 5:**
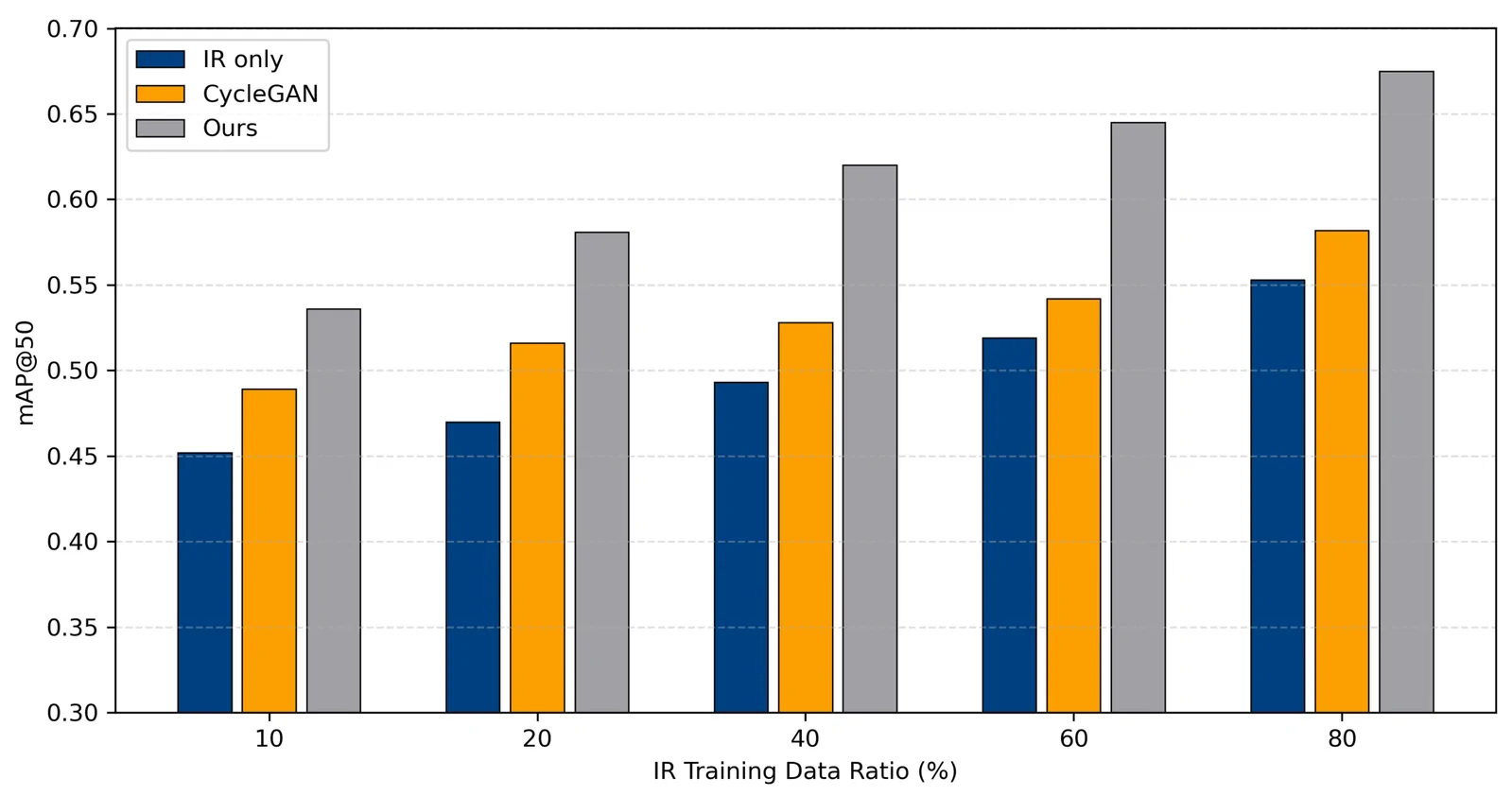
Detection performance on the VEDAI dataset under different proportions of real infrared training data. “IR only” denotes training with real infrared images alone, whereas CycleGAN and SSD-VI denote training with the corresponding synthetic data augmentation.

Figure 5 reports the detection performance under varying degrees of infrared data scarcity, where only 10% to 80% of the real infrared images are available for training. For each data ratio, the detector is trained either with limited real infrared data alone or with an augmented training set expanded with synthesized infrared images generated by different methods. As the amount of real infrared data increases, detection performance improves steadily for all training strategies, reflecting the benefit of additional generated data. Across all data ratios, the proposed method consistently achieves higher mAP@50 than both the IR-only baseline and CycleGAN-based augmentation, demonstrating its robustness across varying levels of data availability. The advantage of the proposed method is particularly evident in low-data regimes. When only 10% to 20% of the infrared training data are available, the proposed method provides a clear performance gain over the baseline, demonstrating its effectiveness in compensating for severe data scarcity. As more real infrared data are introduced, the performance gap gradually narrows, suggesting that the contribution of synthetic data becomes less pronounced once sufficient real data are available.

Table 4 reports the classification performance on the RGB-NIR dataset using different infrared data augmentation methods. The baseline model is trained only on the original infrared data, whereas the other methods incorporate synthesized infrared images to expand the training set. As shown, all augmentation-based methods outperform the baseline, indicating that generated infrared images are beneficial for RGB-NIR classification under limited data. Among the compared approaches, the proposed method achieves the highest accuracy and F1-score, reflecting its superior overall classification performance.

#### 4.2.2. Qualitative Results

**Figure 6:**
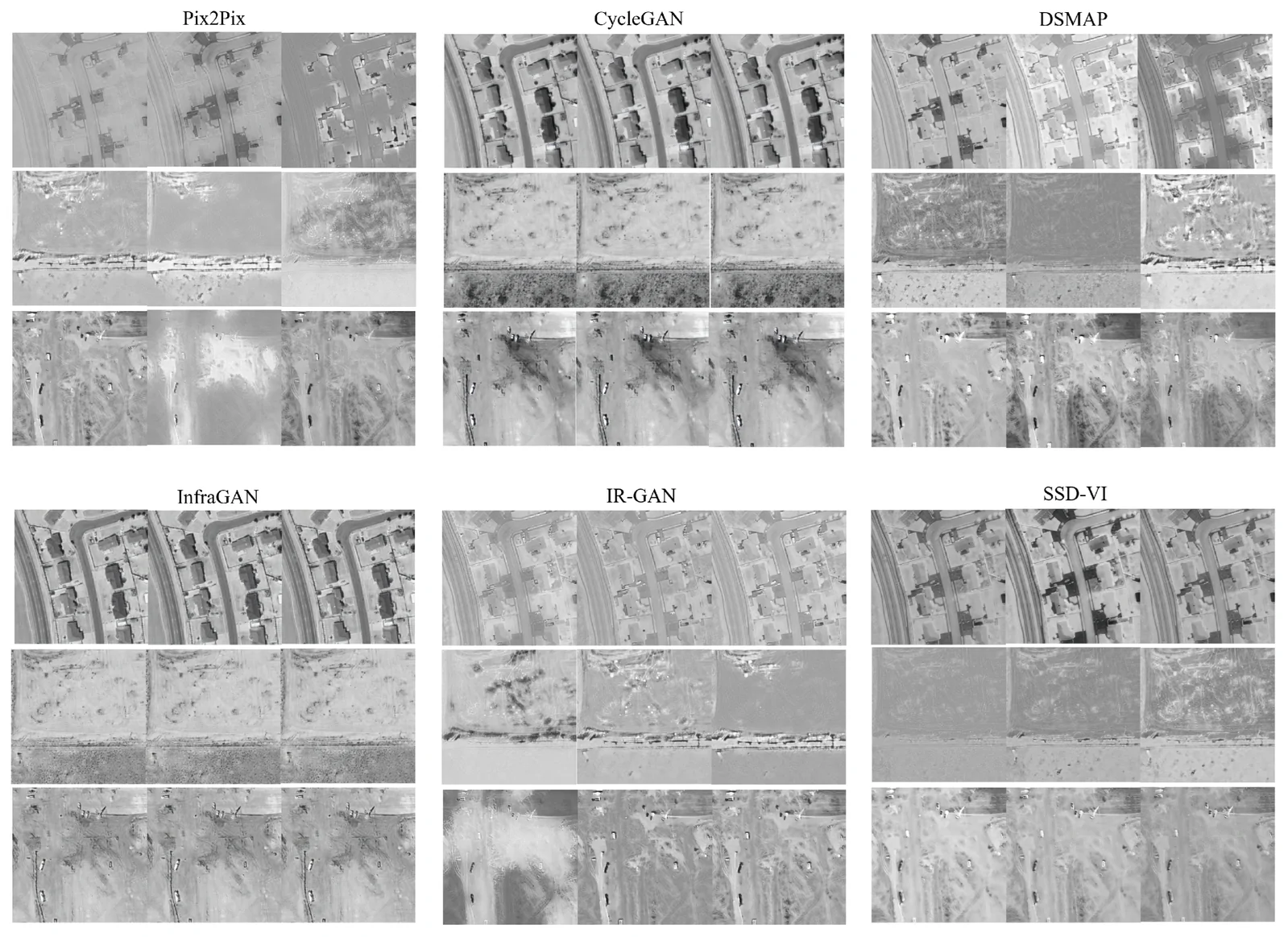
The results of generating infrared images in the RGB-NIR dataset.

**Figure 7:**
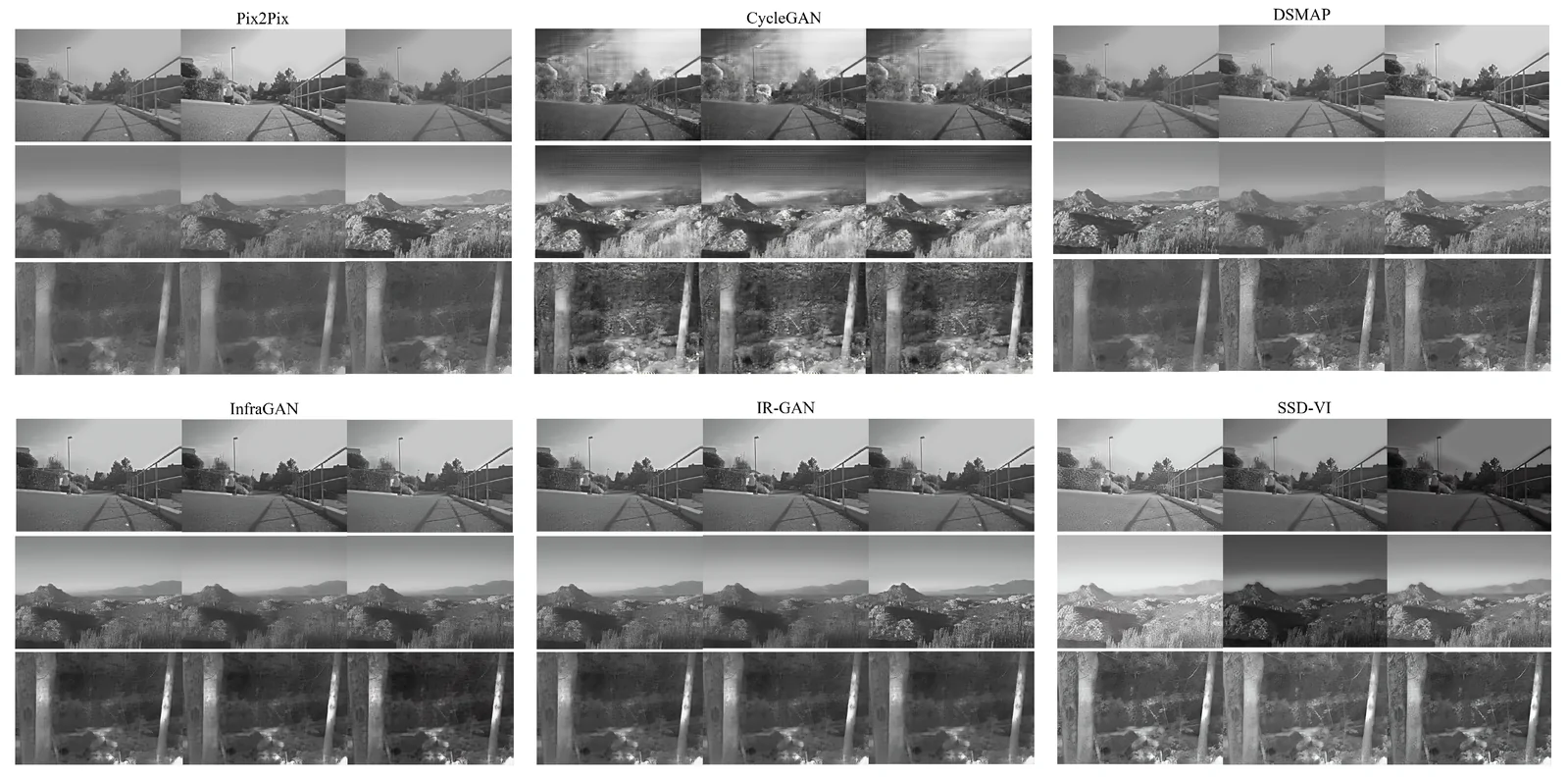
The results of generating infrared images in the VEDAI dataset.

Figure 6 and Figure 7 present qualitative comparisons of optical-to-infrared translation results on the RGB-NIR and VEDAI datasets, respectively. From the visual results, SSD-VI produces infrared images with a more realistic appearance and richer style variations, while preserving the overall semantic structures of the input optical images. In contrast, the competing methods exhibit several noticeable limitations. Pix2Pix and CycleGAN tend to generate infrared images with limited diversity, often producing similar appearances across different inputs. DSMAP improves domain-specific mapping but occasionally introduces structural distortions, particularly around object boundaries. InfraGAN generates visually sharp results in some regions, but the lack of explicit semantic constraints leads to unstable translations across scenes. IR-GAN emphasizes local structural details, yet the generated images often show reduced style variation.

**Figure 8:**
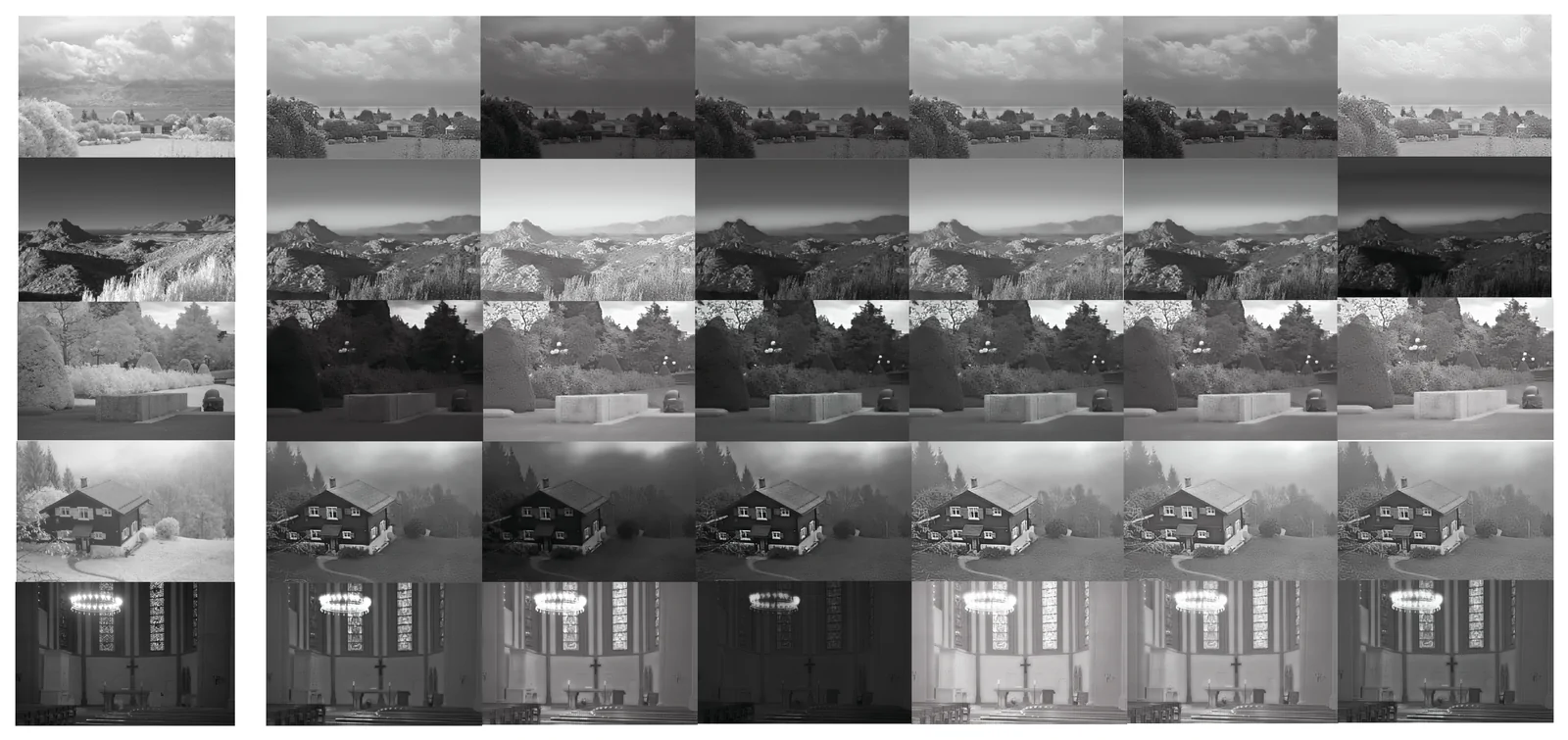
The results of generating infrared images with diverse styles. The first column shows the real infrared images, while the remaining columns are the generated images with diverse styles.

Figure 8 demonstrates the ability of SSD-VI to generate infrared images with diverse style variations from a single optical image. Specifically, the generated infrared images exhibit noticeable differences in intensity distribution and background appearance, while the object structures remain consistent, indicating that SSD-VI can model multiple plausible infrared representations for the same optical scene.

#### 4.2.3. Ablation Study

We conduct ablation experiments on the RGB-NIR dataset to analyze the contributions of the two key components in SSD-VI: the style-semantic disentanglement network (SSD) and the multi-level alignment loss (MAL). The results are reported in Table 6 using realism metrics (FID, KID) and diversity metrics (LPIPS, SSIM).

As shown in the table, introducing SSD alone substantially improves output diversity. Specifically, LPIPS increases from 0.0000 to 0.1817, while SSIM decreases accordingly, indicating that the model no longer produces nearly identical outputs for different style samples. FID and KID are also moderately improved. These results suggest that SSD relaxes the deterministic mapping learned by the baseline and expands the feasible output space by separating recognition-relevant semantic content from infrared-specific style variation. Consequently, multiple infrared appearances can be generated from the same optical input while preserving the underlying target structure. By contrast, MAL without SSD yields a clear improvement in FID and KID but leaves LPIPS at 0.0000. This behavior is expected because MAL acts primarily as a regularizer of the generated output rather than as a source of multimodality. Its semantic-level constraints encourage the translated images to approach the infrared-domain distribution, preserve target geometry, and retain recognition-relevant information. However, when the translator remains deterministic, these constraints can improve fidelity but cannot introduce distinct infrared appearances. The best overall trade-off is achieved when SSD and MAL are used jointly. Their effects are complementary rather than redundant. SSD first enlarges the set of plausible outputs through stochastic style sampling, but this expanded solution space may also contain samples with unrealistic infrared statistics or weakened semantic cues. MAL then constrains this space by suppressing implausible or semantically unreliable solutions while retaining valid style variations. In other words, SSD determines how broadly the model can explore the infrared appearance space, whereas MAL regulates which variations remain consistent with the target domain and downstream recognition objective. This interaction explains why the complete model achieves the lowest FID and KID while maintaining high LPIPS and low inter-output SSIM. The results show that SSD provides the capacity for multimodal generation, whereas MAL converts this capacity into diverse yet reliable infrared samples.

#### 4.2.4. Computational Complexity Analysis

As shown in Table 7, SSD-VI requires 19.25 M parameters and 267 G FLOPs, compared with 11.41 M parameters and 201 G FLOPs for CycleGAN. The additional semantic and style modeling modules increase the parameter count and FLOPs by 68.7% and 32.8%, respectively. Nevertheless, the per-image inference time increases only from 128 ms to 150 ms. This additional cost is incurred during offline data generation and does not affect the deployment-time complexity of the downstream classifier or detector.

### 4.3. Limitations

SSD-VI learns infrared appearance distributions directly from the available training data rather than explicitly modeling the physical image-formation process. Physical factors such as object temperature, surface emissivity, material properties, atmospheric transmission, and sensor-specific radiometric responses are not provided as model inputs. Therefore, although the generated images may exhibit plausible infrared-domain characteristics and improve downstream recognition, their intensity values cannot be interpreted as physically calibrated temperature measurements. In addition, the diversity and realism of the generated infrared appearances depend on the coverage of the training data. Rare thermal states, unseen materials, different sensor responses, or environmental conditions that are insufficiently represented in the training set may not be reproduced accurately. Although the experiments on RGB-NIR and VEDAI demonstrate a certain degree of generalization across datasets, scenes, and tasks, they cannot fully cover the broad variability of infrared sensors and operating conditions. The synthesized images should therefore be regarded as a means of recognition-oriented infrared data augmentation rather than as substitutes for radiometrically calibrated thermal imagery, particularly when infrared variations are governed by physical factors that cannot be reliably inferred from optical observations alone.

## 5. Conclusions

The main conclusions of this study are summarized as follows:

- SSD-VI separates domain-general semantic information from domain-specific appearance statistics, enabling style-conditioned optical-to-infrared generation while preserving recognition-relevant structures.
- Experiments on RGB-NIR and VEDAI show that the generated images improve both classification and detection when infrared training data are limited.
- Future work will aim to enhance the robustness of the framework to unseen scenarios by incorporating domain adaptation techniques and exploring large-scale pretraining paradigms.

## Figures and Tables

**Table 1:** Quantitative results of different methods on the RGB-NIR dataset. Bold values indicate the best-performing results among the compared methods. The downward arrow (↓) indicates that a lower value is better, whereas the upward arrow (↑) indicates that a higher value is better.

Methods	Metrics			
	FID↓	KID↓	LPIPS↑	SSIM↓
Pix2Pix [39]	58.9036	0.0590	0.1775	0.4799
CycleGAN [14]	50.4927	0.0480	0.0000	0.4846
DSMAP [40]	80.5311	0.0705	0.1054	0.5120
InfraGAN [41]	52.4745	0.0663	0.1002	0.4993
IR-GAN [42]	53.3352	0.0596	0.1253	0.5101
**SSD-VI**	**46.5316**	**0.0331**	**0.1859**	**0.3106**

**Table 2:** Quantitative results of different methods on the VEDAI dataset. Bold values indicate the best-performing results among the compared methods. The downward arrow (↓) indicates that a lower value is better, whereas the upward arrow (↑) indicates that a higher value is better.

Methods	Metrics			
	FID↓	KID↓	LPIPS↑	SSIM↓
Pix2Pix [39]	41.9652	0.0090	0.0075	0.9959
CycleGAN [14]	39.5681	0.0073	0.0000	0.9493
DSMAP [40]	60.2154	0.0205	0.0993	0.8787
InfraGAN [41]	38.5431	0.0080	0.0815	0.8086
IR-GAN [42]	33.8557	0.0080	0.0762	0.6564
**SSD-VI**	**30.7096**	**0.0064**	**0.1702**	**0.5312**

**Table 3:** Detection performance comparison on the VEDAI dataset using different detection architectures. Bold values indicate the best-performing results among the compared methods.

Architecture	Metric	Baseline	YOLO-CIR [43]	DOCI-GAN [44]	TC-GAN [45]	Ours
YOLOv8m	mAP@50	0.51	0.55	0.59	0.61	**0.64**
	Precision	0.59	0.62	0.67	0.71	**0.73**
	Recall	0.55	0.57	0.61	0.65	**0.68**
RT-DETR	mAP@50	0.49	0.56	0.58	0.60	**0.63**
	Precision	0.57	0.61	0.66	**0.78**	0.75
	Recall	0.54	0.58	0.62	0.63	**0.66**

**Table 4:** Classification performance on the RGB-NIR dataset. Bold values indicate the best-performing results among the compared methods.

Metric	Baseline	IMT [46]	ILoFGAN [47]	DTAGAN [48]	CWGAN-GP [49]	Ours
Accuracy	0.8337	0.8503	0.8357	0.8806	0.8639	**0.9005**
Recall	0.7866	0.8036	0.7935	**0.8357**	0.8105	0.8203
F1-score	0.8126	0.8296	0.8018	0.8635	0.8402	**0.8857**

**Table 5:** Cross-dataset generalization and resolution robustness of SSD-VI. All evaluations are performed without additional fine-tuning. The downward arrow (↓) indicates that a lower value is better.

Evaluation Setting	Training Setting	Test Setting	FID ↓	KID ↓
Cross-dataset generalization	VEDAI	RGB-NIR	60.0824	0.0612
Resolution robustness	VEDAI, 512×512	VEDAI, 512×512	32.0845	0.0071
Resolution robustness	VEDAI, 512×512	VEDAI, 1024×1024	39.7381	0.0084

**Table 6:** Ablation study of different components on the RGB-NIR dataset. Bold values indicate the best-performing results among the compared methods. The downward arrow (↓) indicates that a lower value is better, whereas the upward arrow (↑) indicates that a higher value is better. In the ablation study, a ✓ indicates that the corresponding component is included in the evaluated model configuration.

Methods	SSD	MAL	FID ↓	KID ↓	LPIPS ↑	SSIM ↓
Baseline			50.4928	0.0480	0.0000	0.4846
w/SSD	✓		49.3025	0.0371	0.1817	0.4653
w/MAL		✓	47.3371	0.0342	0.0000	0.4846
Ours	✓	✓	**46.5316**	**0.0330**	**0.1858**	**0.3106**

**Table 7:** Inference complexity of CycleGAN and SSD-VI at a resolution of 512×512.

Method	Params (M)	FLOPs (G)	Inference Time (ms)
CycleGAN	11.41	201	128
SSD-VI	19.25	267	150

## Data Availability

The original contributions presented in this study are included in the article. Further inquiries can be directed to the corresponding author.
